# Antiplatelet therapy prior to COVID-19 infection impacts on patients mortality: a propensity score-matched cohort study

**DOI:** 10.1038/s41598-024-55407-9

**Published:** 2024-02-28

**Authors:** Mateusz Sokolski, Konrad Reszka, Barbara Adamik, Katarzyna Kilis-Pstrusinska, Weronika Lis, Michał Pomorski, Janusz Sokolowski, Adrian Doroszko, Katarzyna Madziarska, Ewa Anita Jankowska, Marcin Protasiewicz

**Affiliations:** 1 Institute of Heart Disease, Wroclaw Medical University, Borowska 213, 50-556 Wroclaw, Poland; 2 Institute of Heart Disease, University Hospital, Wroclaw, Poland; 3 Department of Anesthesiology and Intensive Therapy, Wroclaw Medical University, Wroclaw, Poland; 4 Clinical Department of Pediatric Nephrology, Wroclaw Medical University, Wroclaw, Poland; 5 Clinical Department of Gynecology and Obstetrics, Wroclaw Medical University, Wroclaw, Poland; 6 Clinical Department of Emergency Medicine, Wroclaw Medical University, Wroclaw, Poland; 7 Clinical Department of Internal and Occupational Diseases, Hypertension and Clinical Oncology, Wroclaw Medical University, Wroclaw, Poland; 8 Department of Nephrology and Transplantation Medicine, Wroclaw Medical University, Wroclaw, Poland

**Keywords:** Cardiovascular biology, Immunological disorders, Drug therapy

## Abstract

One of the major pathomechanisms of COVID-19 is the interplay of hyperinflammation and disruptions in coagulation processes, involving thrombocytes. Antiplatelet therapy (AP) by anti-inflammatory effect and inhibition of platelet aggregation may affect these pathways. The aim of this study was to investigate if AP has an impact on the in-hospital course and medium-term outcomes in hospitalized COVID-19 patients. The study population (2170 COVID-19 patients: mean ± SD age 60 ± 19 years old, 50% male) was divided into a group of 274 patients receiving any AP prior to COVID-19 infection (AP group), and after propensity score matching, a group of 274 patients without previous AP (non-AP group). Patients from the AP group were less frequently hospitalized in the intensive care unit: 9% vs. 15%, 0.55 (0.33–0.94), developed less often shock: 9% vs. 15%, 0.56 (0.33–0.96), and required less aggressive forms of therapy. The AP group had more coronary revascularizations: 5% vs. 1%, 3.48 (2.19–5.55) and strokes/TIA: 5% vs. 1%, 3.63 (1.18–11.2). The bleeding rate was comparable: 7% vs. 7%, 1.06 (0.54–2.06). The patients from the AP group had lower 3-month mortality: 31% vs. 39%, 0.69 (0.51–0.93) and didn’t differ significantly in 6-month mortality: 34% vs. 41%, 0.79 (0.60–1.04). When analyzing the subgroup with a history of myocardial infarction and/or coronary revascularization and/or previous stroke/transient ischemic attack and/or peripheral artery disease, AP had a beneficial effect on both 3-month: 37% vs. 56%, 0.58 (0.40–0.86) and 6-month mortality: 42% vs. 57%, 0.63 (0.44–0.92). Moreover, the favourable effect was highly noticeable in this subgroup where acetylsalicylic acid was continued during hospitalization with reduction of in-hospital: 19% vs. 43%, 0.31 (0.15–0.67), 3-month: 30% vs. 54%, 044 (0.26–0.75) and 6-month mortality: 33% vs. 54%, 0.49 (0.29–0.82) when confronted with the subgroup who had acetylsalicylic acid suspension during hospitalization. The AP may have a beneficial impact on hospital course and mortality in COVID-19 and shouldn’t be discontinued, especially in high-risk patients.

## Introduction

Coronavirus disease 2019 (COVID-19), caused by severe acute respiratory syndrome coronavirus 2 (SARS-CoV-2), has become a global health crisis^1^. SARS-CoV-2 is a single-stranded RNA virus with a high mutation rate^2,3^. Five SARS-CoV-2 variants (alpha, beta, gamma, delta, and omicron) have been identified by WHO as variants of concern. While approximately 80% of SARS-CoV-2 infections are mild to moderate, the clinical presentation and case fatality rate vary depending on the viral variant and comorbidities^4,5^. Thus, the infection fatality rates vary from 0.3 to 5%. The major causes of death are respiratory failure, sepsis/multi-organ failure, cardiac failure, hemorrhage, and renal failure^6–10^. Nevertheless, hypercoagulability and thromboembolic complications became the hallmark of COVID-19^11,12^.

Post-mortem studies have demonstrated the presence of multi-organ thrombosis, even in asymptomatic patients and those on standard thromboprophylaxis^5,13,14^. While fibrin thrombi were observed in small arterial vessels in 87% of the samples analyzed, increased deposition of platelets and megakaryocytes with increased platelet-leukocyte aggregates has also been reported in pulmonary capillaries^15–18^. The incidence rate of thromboembolic events (e.g., venous thromboembolism, pulmonary embolism, stroke, acute coronary syndrome, bowel and limb ischemia) varies between studies. It is highest in critically ill and mechanically ventilated patients and worsens the prognosis^19–21^. The rate of arterial thromboembolism has been estimated at 2.8–8.4%^19^.

SARS-CoV-2 infection can promote thrombosis by several molecular and cellular mechanisms, including not only dysregulation of the renin–angiotensin–aldosterone system (RAAS) and immune response but also platelet and endothelium function alterations^15,20–26^. Platelets play a major role in hemostasis, thrombosis, and inflammatory response^15,27^. COVID-19 is associated with platelet activation, increased tissue factor expression, and the formation of platelet-leukocyte aggregates. Activated platelets interact with dysfunctional endothelium and neutrophils, resulting in thrombogenesis^15^.

Nevertheless, the data on the use of antiplatelet (AP) therapy in COVID-19 patients are conflicting^28–30^. The meta-analysis by Wanting Su et al., which included 34 studies, showed that ASAmay reduce all-cause mortality in patients with COVID-19 by 15–20%^31^.

The complex relationship between SARS-CoV-2 infection and hemostatic dysfunction observed in COVID-19 patients is still not fully understood, and treatment outcomes remain unsatisfactory^32^. While antithrombotic treatment does not appear to protect against morbidity and mortality, there is a need for effective therapy to reduce the incidence of thromboembolic complications and improve outcomes^33–35^.

The aim of the present study was to evaluate the effect of AP treatment prior to COVID-19 infection on the clinical profile, in-hospital course, and short- and medium-term mortality of patients hospitalized with COVID-19. To compare the risk of death among patients with or without prior AP therapy, we conducted a propensity score matching (PSM).

## Results

### Description of the entire COVID-19 cohort and study groups

The clinical characteristic of the 2170 hospitalized patients with COVID-19 is presented in Table 1.

**Table 1:** Baseline characteristic of the entire COVID-19 cohort.

Variables, units	All population, N = 2170
Demographics and co-morbidities	
Age, years	60 ± 19
Male, gender, n (%)	1076 (50)
Body mass index, kg/m^2^	28 ± 5
Co-morbidities	
Arterial hypertension, n (%)	1022 (47)
Diabetes mellitus, n (%)	515 (24)
Hypercholesterolaemia, n (%)	326 (15)
Atrial fibrillation/flutter, n (%)	290 (13)
Previous coronary revascularization, n (%)	154 (7)
Previous myocardial infarction, n (%)	191 (9)
Heart failure, n (%)	255 (13)
Significant valvular heart disease or previous valve heart surgery, n (%)	95 (4)
Peripheral artery disease, n (%)	100 (5)
Previous stroke/transient ischemic attack, n (%)	164 (8)
Chronic kidney disease or/and haemodialysis, n (%)	231 (11)
Asthma, n (%)	85 (4)
Connective tissue disorders, n (%)	51 (2)
Chronic obstructive pulmonary disease, n (%)	75 (3)
Cigarette smoking (previous or current), n (%)	213 (9)
Malignant disease, n (%)	150 (7)
Clinical signs and symptoms at admission	
Cough, n (%)	645 (30)
Dyspnoea, n (%)	919 (42)
Chest pain, n (%)	162 (7)
Haemoptysis, n (%)	15 (1)
Smell dysfunction, n (%)	76 (4)
Taste dysfunction, n (%)	66 (3)
Abdominal pain, n (%)	146 (7)
Diarrhoea, n (%)	127 (6)
Vomiting, n (%)	98 (5)
Body temperature, ℃	37.0 ± 0.9
Heart rate, beats/minute	86 ± 16
Systolic blood pressure, mmHg	132 ± 23
SpO2 on room air, %	92 ± 8
Wheezing, n (%)	219 (10)
Pulmonary congestion, n (%)	367 (17)
Peripheral oedema, n (%)	189 (9)
Treatment applied before hospitalization	
Angiotensin-converting enzyme inhibitors, n (%)	352 (16)
Angiotensin receptor blockers, n (%)	144 (7)
β-blockers, n (%)	533 (25)
Mineralocorticoid receptor antagonists, n (%)	100 (5)
Thiazide or thiazide-like diuretics, n (%)	150 (7)
Vitamin K antagonists, n (%)	47 (2)
Direct oral anticoagulants, n (%)	107 (5)

The numerical variables are presented as mean and standard deviation.

There were 275 (13%) patients receiving antiplatelet treatment, including 258 (94%) patients receiving ASA, 35 (13%) receiving clopidogrel, 3 (1%) ticagrelor and 1 (0.4%) prasugrel. There were 22 (8%) patients on dual antiplatelet therapy.

Based on PSM, the group of 274 patients receiving AP before hospitalization and 274 patients without previous antiplatelet therapy were selected from the study population. Patients were matched 1:1 across each cohort on a propensity score generated by the logistic regression model, adjusting for the following covariates: age, sex, arterial hypertension, heart failure, previous ischemic stroke, renal insufficiency, obesity (body mass index ≥ 30 kg/m^2^), diabetes mellitus.

**Figure 1:**
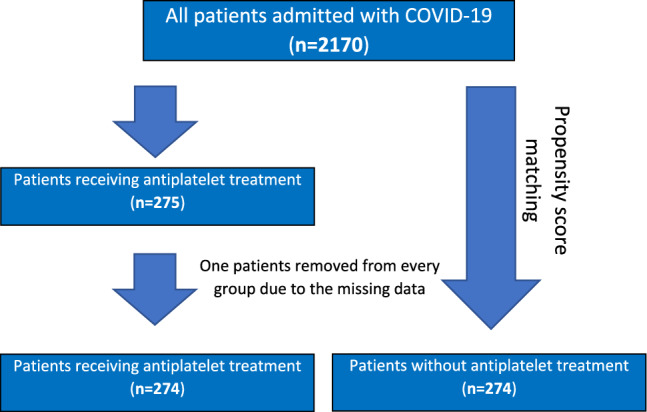
Flowchart of the study population.

Due to the missing data, 2 patients were excluded from the analysis (see Fig. 1 for the flowchart of the study population). The characteristics of two groups of patients (274:274) after PSM are shown in Tables 2 and 3.

**Table 2:** The comparison of the study groups.

Variables, units	AP-group N = 274	Non-AP group N = 274	P
Demographics			
Age, years	71 ± 12	72 ± 13	0.3
Male gender, n (%)	158 (58)	145 (53)	0.3
Body mass index, kg/m^2^	29 ± 6	28 ± 5	0.5
Co-morbidities			
Arterial hypertension, n (%)	223 (81)	233 (85)	0.3
Diabetes mellitus, n (%)	132 (48)	123 (45)	0.4
Hypercholesterolaemia, n (%)	55 (20)	44 (16)	0.9
Atrial fibrillation/flutter, n (%)	56 (20)	84 (31)	0.006
Previous coronary revascularisation, n (%)	80 (29)	29 (11)	< 0.001
Previous myocardial infarction, n (%)	85 (31)	42 (15)	< 0.001
Heart failure, n (%)	84 (31)	87 (32)	0.8
Significant valvular heart disease or previous valve heart surgery, n (%)	27 (10)	26 (10)	0.9
Peripheral artery disease, n (%)	46 (17)	19 (7)	< 0.001
Previous stroke/transient ischemic attack, n (%)	53 (19)	41 (15)	0.2
Chronic kidney disease or/and haemodialysis, n (%)	65 (24)	61 (22)	0.7
Asthma, n (%)	9 (3)	15 (5)	0.2
Chronic obstructive pulmonary disease, n (%)	20 (7)	19 (7)	0.9
Connective tissue disorders, n (%)	13 (5)	5 (2)	0.6
Cigarette smoking (previous or current), n (%)	53 (19)	37 (14)	0.2
Malignant disease, n (%)	27 (10)	23 (8)	0.1
Clinical signs and symptoms at admission			
Cough, n (%)	71 (26)	61 (22)	0.3
Dyspnoea, n (%)	123 (45)	117 (43)	0.6
Chest pain, n (%)	23 (8)	19 (7)	0.5
Haemoptysis, n (%)	3 (1)	3 (1)	0.7
Smell dysfunction, n (%)	9 (3)	4 (1)	0.3
Taste dysfunction, n (%)	10 (4)	4 (1)	0.2
Abdominal pain, n (%)	13 (5)	14 (5)	1.0
Diarrhoea, n (%)	20 (7)	20 (7)	0.9
Vomiting, n (%)	14 (5)	12 (4)	0.8
Body temperature, ℃	36.9 ± 0.8	37.0 ± 0.9	0.6
Heart rate, beats/minute	82 ± 16	85 ± 19	0.1
Systolic blood pressure, mmHg	136 ± 23	134 ± 25	0.5
SpO2 on room air, %	93 ± 6	90 ± 10	0.02
Wheezing, n (%)	42 (15)	44 (16)	0.8
Pulmonary congestion, n (%)	59 (22)	62 (23)	0.8
Peripheral oedema, n (%)	34 (12)	39 (14)	0.5
Treatment applied before hospitalization			
Angiotensin-converting enzyme inhibitors, n (%)	133 (49)	64 (23)	< 0.001
Angiotensin receptor blockers, n (%)	35 (13)	25 (9)	0.2
β-blockers, n (%)	186 (68)	97 (35)	< 0.001
Mineralocorticoid receptor antagonists, n (%)	29 (11)	27 (10)	0.8
Thiazide or thiazide-like diuretics, n (%)	40 (15)	26 (9)	0.07
Vitamin K antagonists, n (%)	10 (4)	12 (4)	0.7
Direct oral anticoagulants, n (%)	16 (6)	18 (7)	0.7
Proton-pump inhibitors, n (%)	89 (32)	44 (16)	< 0.001

The numerical variables are presented as mean and standard deviation.

**Table 3:** Laboratory parameters in the studied groups.

Variables, units	AP-group N = 274	Non-AP group N = 274	P
Morphology			
Leucocytes, 10^3^/µl	7.4 [5.7–10.3]	8.0 [5.7–11.2]	0.3
Lymphocytes, 10^3^/µl	1.0 [0.6–11.4]	0.9 [0.6–1.4]	0.2
Neutrophils, 10^3^/µl	5.4 [3.5–7.9]	5.8 [3.6–8.8]	0.3
Haemoglobin, g/dl	12.5 ± 2.3	12.4 ± 2.5	0.6
Biochemistry			
Sodium, mmol/l	138 ± 6	138 ± 7	0.6
Potassium, mmol/l	4.2 [3.8–4.6]	4.2 [3.6–4.6]	0.5
Urea, mg/dl	48 [34–72]	52 [34–93]	0.3
Creatinine, g/dl	1.1 [0.8–1.6]	1.1 [0.8–1.7]	0.8
Albumin, g/l	3.3 ± 0.6	3.0 ± 0.6	0.005
Uric acid, mg/dl	6.0 [4.7–7.5]	5.7 [4.-7.9]	0.8
Aspat, U/L	33 [24–53]	36 [23–59]	0.7
Alat, U/L	25 [16–43]	27 [17–47]	0.2
Bilirubin, mg/dl	0.6 [0.5–0.9]	0.7 [0.5–1.0]	0.2
GGTP, U/L	38 [22–70]	43[24–83]	0.2
Cardiac biomarkers			
NT-proBNP, pg/ml	3336 [791–13580]	2118 [548–10429]	0.2
Troponin I, ng/l	27 [8–102]	24 [9–92]	0.8
Inflammatory parameters			
CRP at admission, mg/l	44 [10–108]	62 [20–143]	0.02
CRP minimum, mg/l	8 [3–26]	16 [5–44]	< 0.001
CRP maximum, mg/l	90 [33–171]	101 [40–202]	0.08
Procalcitonin at admission, ng/ml	0.09 [0.04–0.27]	0.15 [0.05–0.49]	0.01
Procalcitonin minimum, ng/ml	0.05 [0.03–0.10]	0.06 [0.03–0.170]	0.008
Procalcitonin maximum, ng/ml	0.18 [0.06–0.83]	0.24 [0.08–1.63]	0.04
IL-6 at admission, pg/ml	20 [9–57]	28 [11–61]	0.2
Il-6 minimum, pg/ml	11 [5–28]	18 [7–41]	0.1
Il-6 maximum, pg/ml	22 [10–65]	35 [14–95]	0.1
Ferritin at admission, ng/ml	446 [223–949]	698 [313–1324]	0.004
Ferritin minimum, ng/ml	368 [204–678]	581 [263–1165]	0.006
Ferritin maximum, ng/ml	597 [282–1151]	819 [377–1561]	0.03

The variables are presented as the mean, and standard deviation for normally distributed variables, whereas median with interquartile range (IQ) for non-normally distributed variables.

*AP* antiplatelet treatment, *Aspat* aspartate transaminase, *Alat* alanine transaminase, *GGTP* gamma-glutamyltransferase, *NT-proBNP* N-terminal pro-type brain natriuretic peptide, *CRP* C-reactive protein, *IL-6* interleukin-6.

Both groups did not differ in demographic parameters. Patients from the AP group had more frequent previous coronary revascularization, previous MI, peripheral artery disease (PAD) and less frequently atrial fibrillation/flutter in comparison with the non-AP group. There were no differences in baseline clinical signs and symptoms apart from higher baseline oxygen saturation in room air. The AP group was receiving much more medical treatment than the non-AP group before hospitalization, concerning angiotensin-converting enzyme inhibitors (ACEIs), β-blockers, calcium blockers and loop diuretics. Among laboratory parameters, patients from the AP group had significantly lower levels of inflammatory markers at admission, including CRP, procalcitonin (minimum and maximum values) registered during hospitalization in comparison to the non-AP group. Ferritin as an acute phase marker was also lower in the AP group at admission as well as during hospital stay in comparison to the non-AP group. There were no differences in IL-6 levels.

### The association of AP treatment with the in-hospital course

Patients from the AP group did not differ significantly with respect to the non-AP group in terms of in-hospital mortality 53 (19%) vs. 64 (23%), OR (95% CI) 0.79 (0.52–1.19). However, patients from the AP group developed fewer shocks, were less frequently hospitalized in the intensive care, and the AP group and was less frequently treated with mechanical ventilation. The AP group had more coronary interventions, including angiography, revascularizations, and also suffered more strokes. The bleeding rate was comparable in both groups. The in-hospital course and therapies applied during the hospitalization after PSM are shown in Table 4.

**Table 4:** In-hospital course and therapies applied during the hospitalization in the studied groups.

Variables, units	AP-group N = 274	Non-AP group N = 274	OR	95% CI
In-hospital course				
In-hospital mortality, n (%)	53 (19)	64 (23)	0.79	0.52–1.19
Duration of hospitalisation, days	13 [7–20]	12 [4–20]	1.00	0.99–1.02
Pneumonia, n (%)	151 (55)	159 (58)	0.89	0.63–1.25
Admission at intensive care unit, n (%)	25 (9)	42 (15)	0.55	0.33–0.94
Shock, n (%)	24 (9)	40 (15)	0.56	0.33–0.96
Myocardial infarction, n (%)	9 (3)	6 (2)	1.52	0.53–4.33
Thromboembolic disease, n (%)	5 (2)	11 (4)	0.44	0.15–1.30
Stroke/ transient ischemic attack, n (%)	14 (5)	4 (1.5)	3.63	1.18–11.20
Acute heat failure, n (%)	17 (6)	21 (8)	0.80	0.41–1.55
Bleeding, n (%)	19 (7)	18 (7)	1.06	0.54–2.06
Applied treatment and procedures				
Passive oxygen therapy, n (%)	115 (42)	106 (39)	1.15	0.81–1.61
Non-invasive ventilation, n (%)	34 (12)	19 (7)	1.90	1.05–3.43
Mechanical ventilation, n (%)	23 (8)	44 (16)	0.48	0.28–0.82
Therapy with catecholamines, n (%)	31 (11)	42 (15)	0.70	0.43–1.16
Coronary angiography, n (%)	16 (6)	4 (1.5)	4.19	1.38–12.72
Coronary revascularization, n (%)	15 (5)	3 (1)	3.48	2.19–5.55
Low-molecular-weight heparin, n (%)	190 (69)	178 (65)	1.22	0.85–1.74
Unfractionated heparin, n (%)	14 (5)	23 (8)	0.59	0.30–1.17
Direct oral anticoagulants, n (%)	16 (6)	18 (7)	0.88	0.44–1.77
Vitamin K antagonists, n (%), n (%)	2 (1)	8 (3)	0.24	0.05–1.17
Thrombolytic therapy, n (%)	3 (1)	1 (0.4)	3.02	0.31–29.4
Systemic corticosteroid, n (%)	149 (54)	139 (51)	1.16	0.83–1.62
Tocilizumab, n (%)	1 (0.4)	3 (1.1)	0.35	0.04–3.25
Remdesivir, n (%)	44 (16)	44 (16)	1.0	0.63–1.58
Antibiotic, n (%)	180 (66)	183 (67)	0.95	0.67–1.36

*AP* antiplatelet treatment, *OR* odds ratio, *CI* confidence interval.

### Medium-term outcome

**Figure 2:**
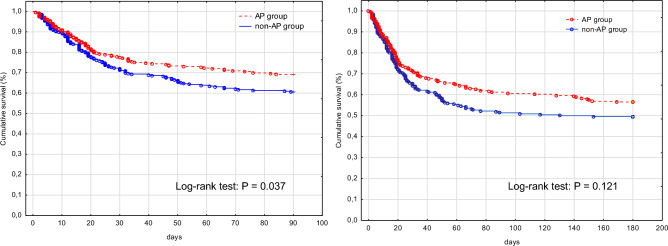
All-cause 3- and 6-month morality.

The groups differ in medium-term outcomes, and patients from the AP group had significantly lower mortality assessed at three months (84 (31%) vs. 108 (39%), HR (95% CI) 0.69 (0.51–0.93)*.* The groups did not differ significantly in terms of 6-month mortality of 94 (34%) vs. 112 (41%), HR (95% CI) 0.79 (0.60–1.04)*.* The Kaplan–Meier analysis with the log-rank test is presented in Fig. 2.

We performed two additional analysis for specific subgroups. First, including only patients with indications for AP therapy (patients with a history of myocardial infarction and/or coronary revascularization and/or previous stroke/transient ischemic attack and/or PAD), the AP group had a lower mortality rate both 3-months: 58 (37%) vs. 47 (56%), HR (95% CI) 0.58 (0.40–0.86) and 6-months: 65 (42%) vs. 48 (57%), HR (95% CI) 0.63 (0.44–0.92), respectively (Table 5).

**Table 5:** Outcomes in the studied subgroups among patients with a history of myocardial infarction and/or coronary revascularization and/or previous stroke/transient ischemic attack and/or peripheral artery disease.

Variables, units	AP-group N = 156	Non-AP group N = 84	OR	95% CI
In-hospital course				
In-hospital mortality, n (%)	37 (24)	27 (32)	0.66	0.36–1.19
Duration of hospitalization, days	13 [7–21]	12 [5–22]	1.00	0.98–1.02
Pneumonia, n (%)	84 (54)	57 (68)	0.55	0.32–0.97
Admission at intensive care unit, n (%)	18 (12)	13 (15)	0.71	0.33–1.54
Shock, n (%)	17 (11)	12 (14)	0.73	0.33–1.63
Myocardial infarction, n (%)	6 (4)	3 (4)	1.08	0.26–4.47
Thromboembolic disease, n (%)	3 (2)	4 (4)	0.67	0.29–1.59
Stroke/transient ischemic attack, n (%)	10 (6)	3 (4)	1.84	0.49–6.96
Acute heat failure, n (%)	12 (7)	9 (11)	0.69	0.28–1.73
Bleeding, n (%)	14 (9)	7 (8)	1.08	0.42–2.82

Variables, units	AP-group N = 156	Non-AP group N = 84	HR	95% CI
Medium-term outcome				
3-months mortality	58 (37)	47 (56)	0.58	0.40–0.86
6-months mortality, n (%)	65 (42)	48 (57)	0.63	0.44–0.92

*AP* antiplatelet treatment, *OR* odds ratio, *CI* confidence interval, *HR* hazard ratio.

Second, divided patients into these who had ASA continuation or ASA suspension during hospitalization (information noted in 193 patients on ASA before admission). The ASA continauation group had a lower mortality rate for in-hospital: 30 (19%) vs. 16 (43%), OR (95% CI) 0.31 (0.15–0.67), 3-months: 47 (30%) vs. 20 (54%), HR (95% CI) 0.44 (0.26–0.75) and 6-months: 52 (33%) vs. 20 (55%), HR (95% CI) 0.49 (0.29–0.82) respectively (Table 6).

**Table 6:** Outcomes in the subgroups among patients with ASA treatment before hospitalization divided into these who had ASA continuation and ASA suspension during hospitalization.

Variables, units	ASA continuation N = 156	ASA suspension N = 37	OR	95% CI
In-hospital course				
In-hospital mortality, n (%)	30 (19)	16 (43)	0.31	0.15–0.67
Duration of hospitalization, days	15 [10–21]	13 [7–25]	0.99	0.97–1.02
Pneumonia, n (%)	94 (60)	27 (73)	0.56	0.26–1.25
Admission at intensive care unit, n (%)	18 (12)	16 (43)	0.17	0.08–0.39
Shock, n (%)	19 (12)	14 (38)	0.23	0.10–0.52
Myocardial infarction, n (%)	5 (4)	3 (8)	0.38	0.08–1.66
Thromboembolic disease, n (%)	4 (3)	3 (8)	0.22	0.04–1.17
Stroke/transient ischemic attack, n (%)	8 (5)	0 (0)	–	–
Acute heat failure, n (%)	8 (5)	4 (11)	0.45	0.13–1.58
Bleeding, n (%)	9 (6)	3 (8)	0.69	0.18–2.72
Medium-term outcome				
3-months mortality, n (%)	47 (30)	20 (54)	0.44	0.26–0.75
6-months mortality, n (%)	52 (33)	20 (54)	0.49	0.29–0.82

*ASA* acetylsalicylic acid, *OR* odds ratio, *CI* confidence interval, *HR* hazard ratio.

**Figure 3:**
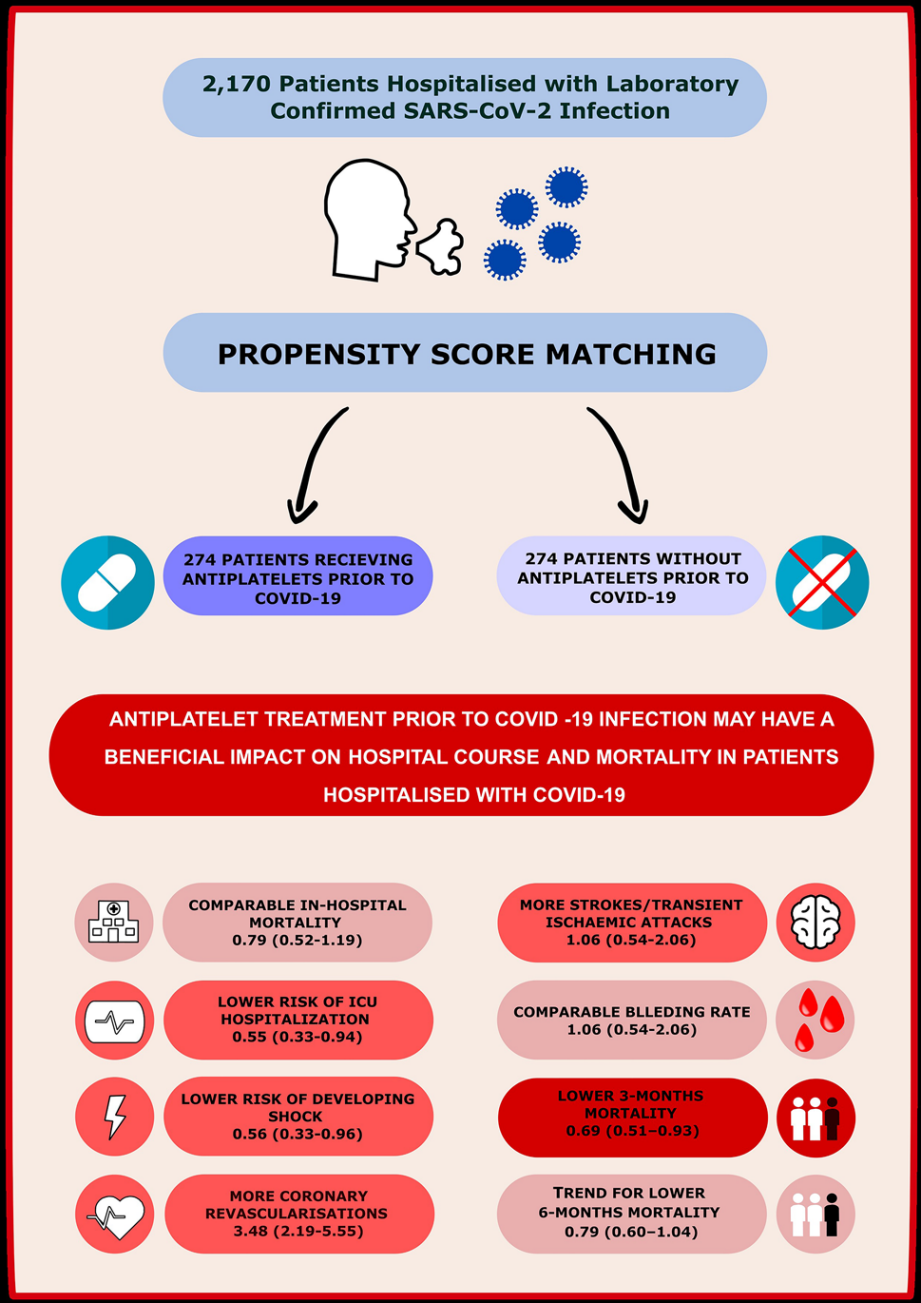
Study summary.

After the adjustment of variables that appeared to be significant predictors in univariate Cox analysis, older age, male gender, previous MI, low oxygen saturation on room air at admission and systemic corticosteroid appeared to be predictors of higher risk for 3-month mortality in multivariable analysis, AP was associated with lower risk for 3-month mortality (Table 7). The graphical summary of the study consist Fig. 3.

**Table 7:** Predictors of 3-month mortality—univariable and multivariable model.

Variables	Units	Univariable model	Multivariable model; Chi^2^ (*p*): 112 (< 0.001)	
		HR (95% CI)	HR (95% CI)	Wald’s statistics
Age	5 years	1.22 (1.14–1.30)	1.25 (1.16–1.35)	33
Male gender	yes/no	1.52 (1.13–2.03)	1.66 (1.21–2.28)	10
Body mass index	1 kg/m^2^	1.01 (0.96–1.05)		
Arterial hypertension	Yes/no	1.44 (0.95–2.20)		
Atrial fibrillation/flutter	Yes/no	1.95 (1.45–2.61)	1.23 (0.84–1.79)	1.2
Diabetes mellitus	Yes/no	1.50 (1.13–2.00)	1.31 (0.98–1.75)	3.3
Previous myocardial infarction	Yes/no	2.21 (1.64–2.97)	1.83 (1.30–2.57)	12
Heart failure	Yes/no	2.23 (1.68–2.96)	1.27 (0.91–1.79)	2.0
Previous stroke/transient ischemic attack	Yes/no	1.41 (1.00–1.99)	1.07 (0.74–1.55)	0.1
Chronic kidney disease or/and haemodialysis	Yes/no	1.77 (1.31–2.39)	1.16 (0.84–1.62)	0.8
Chronic obstructive pulmonary disease	Yes/no	1.76 (1.12–2.77)	1.00 (0.62–1.63)	0.0001
SpO2 on room air, %	%	0.96 (0.94–0.98)	0.98 (0.96–1.00)	4.2
Peripheral artery disease	Yes/no	1.32 (0.88–1.99)		
Malignant disease	Yes/no	1.14 (0.72–1.81)		
Angiotensin-converting enzyme inhibitors	Yes/no	1.20 (0.89–1.60)		
β-blockers	Yes/no	1.19 (0.90–1.58)		
Antiplatelet treatment	Yes/no	0.74 (0.56–0.98)	0.71 (0.53–0.96)	5
Anticoagulation	Yes/no	1.39 (1.02–1.89)	1.02 (0.71–1.46)	0.009
Tocilizumab	Yes/no	1.71 (0.42–6.89)		
Remdesivir	Yes/no	1.00 (0.67–1.47)		
Systemic corticosteroid	Yes/no	1.42 (1.06–1.89)	1.51 (1.12–2.05)	7.2

*HR* hazard ratio, *CI* confidence interval,

## Discussion

The results of our study show that AP may have beneficial impact on the in-hospital course and medium-term mortality of patients hospitalized with COVID-19. Moreover, AP did not increase the number of hemorrhagic complications. Importantly we have also found significantly lower inflammatory markers in the AP group, suggesting a potential mechanism in reducing the excessive inflammatory response, underlying the pathophysiology of COVID-19.

The study group consisted of COVID-19 patients hospitalized between 2020 and 2021. These patients faced more aggressive variants of the virus coupled with the absence of a vaccination program at that time. It should be emphasized, that the studied subgroups are characterized by a high number of comorbidities and risk factors, which probably largely determine a worse prognosis, when affected by COVID-19. Hence, AP in high cardiovascular risk groups could offer significant benefits and should be considered in COVID-19 even with more benign viral variants.

The more frequent coronary angiography and revascularization in the AP group may be linked to a higher incidence of pre-hospitalization coronary problems. Still, despite the greater number of comorbidities and vascular events during hospitalization, the overall prognosis was better in the AP group. A doubly robust estimation, with potential confounders, including medical treatment, also showed potential benefits of AP treatment.

Coronary artery disease (CAD) or PAD constitutes an indication for long-term AP therapy as secondary prevention. There were also patients with a history of MI in the non-AP group. The lack of AP treatment or its disconituation can be explained by the use of anticoagulation, according to the European guidelines, which recommend them as the only treatment usually after 12 months since MI^36^. Anticoagulation has been shown to be more effective in preventing thromboembolic events in atrial fibrillation which may also explain the lower number of ischemic strokes and TIA in the non-AP group. Interestingly, a recent study reported that combination of therapeutic dose of heparin with AP did not improve outcome compared with therapeutic doses of heparin alone^29^.

Although AP treatment is a recognized risk factor for major bleeding, especially in long-term observation, in older patients, and without the routine proton-pump inhibitors (PPI) use^37–39^, we observed no significant differences in hemorrhagic complications between the groups. The use of PPI, given to one-third of AP-treated patients, may be a contributing factor.

Patients in the AP group were more frequently treated with ACEIs and β-blockers. The result of our study are consistent with large single-center registry in Poland, which found that treatment with ACEIs/ARBs, β-blockers, statins, or AP was associated with lower risk of in-hospital death in patients with COVID-19. Authors did not however studied the effect of AP on medium-term prognosis^38^.

The importance of the inflammatory repsonce in the pathogenesis of cardiac commplications in the course of COVID-19 is well established in the have multisystem inflammatory syndrome in children, which is late immune-mediated complication occurring after SARS-CoV-2 infection^40,41^. Thus, beneficial impact of AP on hospital course and mortality in COVID-19 can be explained by anti-inflammatory effects of AP agents^15–18,42^.

It was proven, that P2Y12 inhibitors (i.e. clopidgrel, ticagrelor, prasugrel) may reduce the platelet-related release of pro-inflammatory markers and the formation of platelet-leukocyte aggregates^15–18,43^. They can also increase endothelial nitric oxide bioavailability and reduce oxidative stress in patients with CAD^44^. ASA exerts not only anti-inflammatory effects but may have some antiviral activity on the level of viral ribonucleic acids^45^. In some studies, the pre-admission treatment with ASA was associated with better in-hospital outcomes and a reduced need for respiratory support^46^.

### Limitations

The study is a retrospective analysis of a single-center cohort, which may limit its evidence. Despite the PSM, it is possible that some factors not included in the model could impact the outcomes. While the analysis was based on the data about AP prior to the hospitalization, the data about the duration of the treatment prior COVID-19 and in-hospital treatment were not fully gathered. In the majority of cases the treatment during hospitalization continued the one applied before. Patients were also discharged home with similar medication introduced before admission. Therefore the AP effect could also be the effect of ongoing treatment not only before admission. There were no differences in the use of anticoagulation drugs during hospitalization. Undoubtedly, further prospective studies are needed to verify the clinical value of AP treatment in COVID-19 hospitalized patients and in order to create an optimal medical strategy for SARS-CoV-2 and SARS-CoV-like future infections.

The results of our study show that AP prior to COVID-19 infection may have a beneficial impact on the in-hospital course, mainly driven by the reduction of respiratory complications and intensive care unit admissions. AP may also influence medium-term mortality in COVID-19 and shouldn’t be discontinued, especially in the high-risk patients.

## Materials and methods

### Study population

We included consecutive patients ≥ 18 years, hospitalized in the University Hospital, Wroclaw (Poland), between March 2020 and May 2021, with COVID-19 confirmed by polymerase chain reaction testing of a nasopharyngeal sample or a positive blood antigen test. The study cohort was divided into two groups according to AP status.

- Patients receiving any antiplatelet treatment (acetylsalicylic acid (ASA) and/or clopidogrel/ticagrelor/prasugrel) prior to COVID-19 infection (AP group)
- Matched patients without antiplatelet treatment (non-AP group).

### Data sources

Analyzed variables (demographics, laboratory measurements, comorbidities) were retrospectively collected from the electronic hospital system. The study protocol for the COLOS (COronavirus in the LOwer Silesia registry) study has been approved by the Institutional Review Board and Ethics Committee at the Wroclaw Medical University, Wroclaw, Poland (No.: KB-444/2021). The Bioethics Committee approved the publication of fully anonymized data. Written informed consent to participate in the study was waived to limit unnecessary contact and transmission of the virus. All methods were performed in accordance with the relevant guidelines and regulations. Patients who survived were followed up by telephone contact after three and six months. The patients who were contacted for their data regarding outcome gave the oral informed consent at discharge. Information regarding medium-term outcomes was obtained directly from patients, their relatives, the hospital system, and from Government General Registry Office.

### Endpoints and outcomes

The medium-term clinical outcomes were defined as 3-month, 6-month all-cause mortality. Data regarding in-hospital outcomes were also collected: in-hospital mortality, duration of hospitalization, pneumonia, admission to intensive care unit (ICU), shock, myocardial infarction (MI), thromboembolic disease, stroke/ transient ischemic attacks, acute heart failure, and all-type symptomatic bleeding. We have also analyzed the applied treatment and procedures during hospitalization, including ventilation type: passive oxygen therapy, non-invasive ventilation (high-flow nasal cannula, continuous positive airway pressure, biphasic positive airway pressure), mechanical ventilation, and the need for intubation and invasive mechanical ventilation, therapy with catecholamines,, coronary angiography and revascularization and medical treatment used.

### Statistics

Categorical variables were presented as numbers and percentages, the numerical variables as the mean and standard deviation for normally distributed variables, whereas median with interquartile range (IQ) for non-normally distributed variables. The Shapiro–Wilk test was used to verify the distribution of continuous variables, and the Mann–Whitney U test was applied for group comparison. The chi-square test or Fisher’s exact test was used to compare qualitative variables.

PSM was performed using the match function of the MatchIt R package. The function parameters were set to the logistic regression model, with adjustments for the covariates. Patients were matched using the nearest neighbor technique. Balanced pairs of patients in relation to variables that could impact the outcome were selected from the entire population of 2168 patients.

The association of AP treatment with the in-hospital course was tested with logistic regression model. Kaplan–Meier curves with time to death were constructed to estimate the effect of antiplatelet treatment on all-cause 90, and 180-day mortality. Differences in survival rates were tested with the log-rank test. For the doubly robust estimation, the associations between survival and potential clinical confounder, including other medical treatments, were tested using the univariable and multivariable Cox proportional hazard regression model. The univariable model was performed on the variables (demographics, co-morbidities, clinical signs and symptoms at admission and treatment applied before and during hospitalization) that showed significant association with mortality in COVID-19 in previous studies (age, gender, BMI), which differed between the AP and non-AP groups and which were not interdependent. The multivariable model included variables that were statistically significant and associated with univariable models.

All analyses were performed using Statistica v.13.3 (TIBCO Software Inc., Palo Alto, CA, USA) except PSM, which was performed with the MatchIt R package. The P values < 0.05 were considered statistically significant.

## Data Availability

The datasets analyzed during the current study are available from the corresponding author on reasonable request.

## References

[1] World Health Organisation. WHO director-general’s opening remarks at the media briefing on COVID-19, https://www.who.int/director-general/speeches/detail/who-director-general-s-opening-remarks-at-the-media-briefing-on-covid-19---11-march-2020 (2020).

[2] Malik JA (2022). The SARS-CoV-2 mutations versus vaccine effectiveness: New opportunities to new challenges. J. Infect. Public Health..

[3] Jin Y (2020). Virology, epidemiology, pathogenesis, and control of COVID-19. Viruses..

[4] Fernandes Q (2022). Emerging COVID-19 variants and their impact on SARS-CoV-2 diagnosis, therapeutics and vaccines. Ann. Med..

[5] Zuin M (2022). Prevalence of acute pulmonary embolism at autopsy in patients with COVID-19. Am. J. Cardiol..

[6] Streeck H (2020). Infection fatality rate of SARS-CoV2 in a super-spreading event in Germany. Nat. Commun..

[7] Yang X (2020). Clinical course and outcomes of critically ill patients with SARS-CoV-2 pneumonia in Wuhan, China: A single-centered, retrospective, observational study. Lancet Respir. Med..

[8] Zhang, B. *et al*. Clinical characteristics of 82 cases of death from COVID-19. *PLoS One.* **15**, e0235458. 10.1371/journal.pone.0235458 (2020). 10.1371/journal.pone.0235458 PMC7347130 32645044

[9] Sokolski M (2021). Heart failure in COVID-19: The multicentre, multinational PCHF-COVICAV registry. ESC Heart Fail..

[10] Sokolski M (2022). History of heart failure in patients hospitalized due to COVID-19: Relevant factor of in-hospital complications and all-cause mortality up to six months. J. Clin. Med..

[11] Iba T, Connors JM, Levy JH (2020). The coagulopathy, endotheliopathy, and vasculitis of COVID-19. Inflamm. Res..

[12] Litvinov RI (2021). Altered platelet and coagulation function in moderate-to-severe COVID-19. Sci. Rep..

[13] Ackermann M (2020). Pulmonary vascular endothelialitis, thrombosis, and angiogenesis in covid-19. N. Engl. J. Med..

[14] Rapkiewicz, A.V. *et al*. Megakaryocytes and platelet-fibrin thrombi characterize multi-organ thrombosis at autopsy in COVID-19: A case series. *EClinicalMedicine.* **24**, 100434. 10.1016/j.eclinm.2020.100434 (2020). 10.1016/j.eclinm.2020.100434 PMC7316051 32766543

[15] Brambilla M, Canzano P, Becchetti A, Tremoli E, Camera M (2022). Alterations in platelets during SARS-CoV-2 infection. Platelets..

[16] Dwiputra Hernugrahanto, K. *et al*. Thromboembolic involvement and its possible pathogenesis in COVID-19 mortality: Lesson from post-mortem reports. *Eur. Rev. Med. Pharmacol. Sci.* **25**, 1670–1679 (2021). 10.26355/eurrev_202102_24878 33629337

[17] McMullen PD (2021). A descriptive and quantitative immunohistochemical study demonstrating a spectrum of platelet recruitment patterns across pulmonary infections including COVID-19. Am. J. Clin. Pathol..

[18] Carsana L (2020). Pulmonary post-mortem findings in a series of COVID-19 cases from northern Italy: A two-centre descriptive study. Lancet Infect. Dis..

[19] Mondal S, Quintili AL, Karamchandani K, Bose S (2020). Thromboembolic disease in COVID-19 patients: A brief narrative review. J. Intensive Care..

[20] Ali MAM, Spinler SA (2021). COVID-19 and thrombosis: From bench to bedside. Trends Cardiovasc. Med..

[21] Case BC (2021). Comparison of outcomes in patients with COVID-19 and thrombosis versus those without thrombosis. Am. J. Cardiol..

[22] Levi M, van der Poll T (2010). Inflammation and coagulation. Crit. Care Med..

[23] Sokolski M (2020). Cardiac emergencies during the coronavirus disease 2019 pandemic in the light of the current evidence. Kardiol. Pol..

[24] Taus F (2020). Platelets promote thromboinflammation in SARS-CoV-2 pneumonia. Arterioscler. Thromb. Vasc. Biol..

[25] Rola P (2022). Invasive assessment of coronary microvascular dysfunction in patients with long COVID: Outcomes of a pilot study. Kardiol. Pol..

[26] Patoulias D, Dimosiari A, Michailidis T (2023). Coronary microvascular dysfunction in the context of long COVID-19: What is the effect of anti-inflammatory treatment?. Kardiol. Pol..

[27] Rayes J, Bourne JH, Brill A, Watson SP (2019). The dual role of platelet-innate immune cell interactions in thrombo-inflammation. Res. Pract. Thromb. Haemost..

[28] Salah HM, Mehta JL (2021). Meta-analysis of the effect of aspirin on mortality in COVID-19. Am. J. Cardiol..

[29] Berger JS (2022). Effect of P2Y12 inhibitors on survival free of organ support among non-critically ill hospitalized patients with COVID-19: a randomized clinical trial. JAMA..

[30] Matli, K. *et al*. Combined anticoagulant and antiplatelet therapy is associated with an improved outcome in hospitalised patients with COVID-19: a propensity matched cohort study. *Open Heart*. **8**, e001785. 10.1136/openhrt-2021-001785 (2021). 10.1136/openhrt-2021-001785 PMC8493601 34611018

[31] Su, W. *et al*. Associations between the use of aspirin or other antiplatelet drugs and all-cause mortality among patients with COVID-19: A meta-analysis. *Front. Pharmacol.* **13**, 989903. 10.3389/fphar.2022.989903 (2022). 10.3389/fphar.2022.989903 PMC9581252 36278186

[32] Di Minno A, Ambrosino P, Calcaterra I, Di Minno MND (2020). COVID-19 and venous thromboembolism: A meta-analysis of literature studies. Semin. Thromb. Hemost..

[33] Toubasi AA (2022). Effect on morbidity and mortality of direct oral anticoagulants in patients with COVID-19. Am. J. Cardiol..

[34] Stone, G. W. *et al*. FREEDOM COVID anticoagulation strategy randomized trial investigators. randomized trial of anticoagulation strategies for noncritically ill patients hospitalized with COVID-19. *J. Am. Coll. Cardiol.* **81**, 1747–1762 (2023). 10.1016/j.jacc.2023.02.041 PMC9987252 36889611

[35] Protasiewicz M (2022). Anticoagulation prior to COVID-19 infection has no impact on 6 months mortality: A propensity score-matched cohort study. J. Clin. Med..

[36] Neumann FJ (2019). 2018 ESC/EACTS Guidelines on myocardial revascularization. EuroIntervention..

[37] Protasiewicz M, Szymkiewicz P, Kuliczkowski W, Mysiak A, Witkiewicz W (2013). Modern antiplatelet therapy—opportunities and risks. Adv. Clin. Exp. Med..

[38] Li, L., Geraghty, O.C., Mehta, Z., Rothwell, P.M.; Oxford Vascular Study. Age-specific risks, severity, time course, and outcome of bleeding on long-term antiplatelet treatment after vascular events: a population-based cohort study. *Lancet*. **390**, 490–499 (2017). 10.1016/S0140-6736(17)30770-5 PMC5537194 28622955

[39] Fischer, A.L. *et al*. Antiplatelet agents for the treatment of adults with COVID-19. *Cochrane Database Syst. Rev.* **7**, CD015078. 10.1002/14651858.CD015078 (2023). 10.1002/14651858.CD015078 PMC10368416 37489818

[40] Ludwikowska KM, Moksud N, Tracewski P, Sokolski M, Szenborn L (2023). Cardiac involvement in patients with multisystem inflammatory syndrome in children (MIS-C) in Poland. Biomedicines..

[41] Kumar R, Rivkin MJ, Raffini L (2023). Thrombotic complications in children with Coronavirus disease 2019 and Multisystem Inflammatory Syndrome of Childhood. J. Thromb. Haemost..

[42] Liao Y (2023). Identification of potential new COVID-19 treatments via RWD-driven drug repurposing. Sci. Rep..

[43] Thomas MR, Storey RF (2015). Effect of P2Y12 inhibitors on inflammation and immunity. Thromb. Haemost..

[44] Heitzer T (2006). Clopidogrel improves systemic endothelial nitric oxide bioavailability in patients with coronary artery disease: Evidence for antioxidant and antiinflammatory effects. Arterioscler. Thromb. Vasc. Biol..

[45] Bianconi V (2020). Is acetylsalicylic acid a safe and potentially useful choice for adult patients with COVID-19?. Drugs..

[46] Sisinni A (2021). Pre-admission acetylsalicylic acid therapy and impact on in-hospital outcome in COVID-19 patients: The ASA-CARE study. Int. J. Cardiol..

